# Bacterial growth, detachment and cell size control on polyethylene terephthalate surfaces

**DOI:** 10.1038/srep15159

**Published:** 2015-10-14

**Authors:** Liyun Wang, Daming Fan, Wei Chen, Eugene M. Terentjev

**Affiliations:** 1 School of Food Science and Technology, Jiangnan University, Wuxi 214122, China; 2 Cavendish Laboratory, University of Cambridge, J. J. Thomson Avenue, Cambridge CB3 0HE, UK

## Abstract

In medicine and food industry, bacterial colonisation on surfaces is a common cause of infections and severe illnesses. However, the detailed quantitative information about the dynamics and the mechanisms involved in bacterial proliferation on solid substrates is still lacking. In this study we investigated the adhesion and detachment, the individual growth and colonisation, and the cell size control of *Escherichia coli* (*E. coli*) MG1655 on polyethylene terephthalate (PET) surfaces. The results show that the bacterial growth curve on PET exhibits the distinct lag and log phases, but the generation time is more than twice longer than in bulk medium. Single cells in the lag phase are more likely to detach than clustered ones in the log phase; clustered bacteria in micro-colonies have stronger adhesive bonds with surfaces and their neighbours with the progressing colonisation. We show that the cell size is under the density-dependent pathway control: when the adherent cells are at low density, the culture medium is responsible for coordinating cell division and cell size; when the clustered cells are at high population density, we demonstrate that the effect of quorum sensing causes the cell size decrease as the cell density on surfaces increases.

A significant proportion of food-industrial equipment and medical devices become the focus of biofilm infections, causing lethal illnesses and heavy costs in maintenance^1^,^2^. Hence, biofilm has been of considerable interest in medical and food industry, as well as in academic research^1^,^3^. Biofilms are complex communities of bacteria that develop on solid surfaces and enclose themselves in an extracellular polymeric substance (EPS) matrix^4^,^5^, which acts to protect bacteria from antibiotics, disinfectants and environmental insults^6^,^7^,^8^. The formation of biofilm mainly proceeds in three steps: 1) the initial adhesion of bacterial cells to surfaces, which has been widely studied^9^,^10^,^11^. This step is strongly influenced by the topographical and chemical features of the surface. Bacterial extracellular appendages such as flagella and fimbriae also play an important role in the attachment process^10^,^12^. The next stage is: 2) proliferation of the adherent bacteria and synthesis of the EPS matrix^6^,^13^, followed by the final step: 3) biofilm maturation followed by detachment and lysis of part of biofilms, thus releasing free bacteria to begin another biofilm growth cycle^10^,^11^. Over the last decade, much progress has been achieved in studying the steps (2) and (3). For instance, biochemical and genetic methods were used to study the production of EPS and biofilm-generating systems^8^; the microscopic methods have helped to determine the overall morphology and detachment of biofilms^1^,^14^,^15^,^16^. However, the studies of dynamics of bacterial proliferation after adhesion in the second step are still lacking and, in general, the bacterial colonisation on substrates is yet to be better understood.

The conventional population growth in bulk liquid medium is a very well-studied subject^17^. Figure 1 illustrates the generic four-phase pattern of a standard bacterial population growth, known as the ‘growth curve’, with the population in the ‘log’ phase growing exponentially. That is, the increment of the cell number is in proportion to the current population: *dn/dt *= *αn*, where *n* is the number of bacteria in culture medium at time *t*, and α is a constant known as the ‘specific growth rate’. Integration gives the well-known logarithmic relation for growth: ln(*n/n*_*0*_ = *α*(*t* − *t*_0_) or *n* = *n*_*0*_*e*^*α*(*t* − *t*_0_)^, where *n*_0_ is the initial number of bacteria at a time *t*_0_ when the lag phase ends or the log phase begins. Hence, the generation time or ‘doubling time’ (τ) of cell population is given by: *τ *= ln2/*α*. In this study we are interested in what bacterial growth curve on solid surfaces would be like: whether distinct phases could also be observed, and if so – what the growth rate and generation time would be?

Coordination between cell growth and division ensures an appropriate bacterial cell size for a given environmental condition and developmental fate^18^. Based on the dynamics of bacterial growth and division on surfaces with unlimited external nutrient supply, we also discuss the changes in the cell size as the population grows and attempt to construct a model for the control of cell size under such conditions. The bacterial size control during periods of steady growth in the bulk culture medium has been extensively studied in the last decade^18^,^19^. It is established that FtsZ, the major cytoskeletal protein during binary fission, plays a vital role in the cell size control^20^. When the accumulation of FtsZ in the cell reaches a critical level, this protein self-assembles to form a contractile ring structure (the FtsZ ring or Z-ring) under the membrane at the future division site (the centre of the cell), thereby triggering cell division. It was determined that the regulation of the FtsZ assembly controls the timing of cell division, subsequently altering the cell size^21^. More recently, a nutrient-dependent metabolic pathway has been widely studied to describe the mechanism of cell size regulation in response to the components of culture medium^19^,^22^. When bacteria are grown in a rich medium like Luria Bertani (LB) broth, an FtsZ inhibitor would be activated to suppress the FtsZ assembly, resulting in the cell size having to increase in order to supply sufficient assembly-competent FtsZ; in contrast, when bacteria grow in a nutrient-poor source with a slow growth rate, the inhibitor would be in a low activation regime and bacterial cells subsequently live in a short-sized life.

We have investigated the details of bacterial adhesion on PET surfaces in an earlier publication^23^; this study deals with the growth and detachment of the adherent *Escherichia coli* MG1655 cells on PET in the presence of unlimited external nutrient sources. PET is the substrate of choice since it is ubiquitous in food packaging^24^ and widely used in cardiovascular implants (e.g. artificial heart valve sewing rings and artificial blood vessels) due to its excellent physicochemical properties: good mechanical strength, stability in the presence of body fluids, and relatively high biocompatibility^25^,^26^,^27^. By carefully monitoring the number and size of bacteria on the surface over time, while constantly refreshing the external medium to maintain the constant environment, we determine the “growth curve on a substrate” and also isolate the mechanism of size control of the adhered cells. Our study provides evidence regarding of the process of infection of surfaces in a nutritious environment (e.g. milk or blood) in situations in which microorganisms are not completely removed from food-contact surfaces or in which medical implants become contaminated before and during surgery.

## Methods

### Substrate

Homogeneous PET films with a thickness of 0.35 mm and low roughness (Ra = 5 ± 0.2 nm) were purchased from Goodfellow Cambridge Ltd. (Huntingdon, UK). The films were cut into many identical small pieces (21.5 mm × 8 mm) and cleaned ultrasonically in absolute ethanol for 15 min and then in deionised purified water for 15 min. They were then dried with nitrogen.

### Bacterial stain and culture

In order to prepare a required active synchronised bacterial suspension for our study, we followed the standard procedure: bacterial colonies of *E. coli* MG1655 were stored at 4 °C. A single colony was inoculated into a test tube containing 5 mL of liquid LB culture medium and grown overnight at 37 °C, with gentle shaking at 200 rpm. A 100 μL of this culture was transferred into a fresh tube of 5 mL LB medium and incubated with shaking until the stationary phase was reached (12 to 14 h) to obtain the eventual microbial suspension, which contained ca. 3 × 10^9^ colony forming units per mL (CFU/mL).

### Measurement procedure

Figure 2 illustrates the overall procedure for the measurement of *E. coli* growth on and detachment from the PET surfaces in our study. Because continuous recruitment of new cells from the incubation medium onto surface may contribute to the count of bacteria on the substrates, the external medium in which the PET surfaces were incubated was refreshed each hour. Our experimental procedure has followed the sequence of steps:

Process (I) – a large set of identical clean PET surfaces were sterilised with 70% ethanol for 15 min and rinsed thrice with sterile water and then with LB medium. Each substrate sample was then placed vertically into a test tube (diameter, 2.2 cm) containing stationary-phase synchronised bacterial cells suspended in LB culture at 37 °C for 1 h, to seed bacterial cells on the surfaces (i.e. to carry out the initial bacterial adhesion).

Process (II) – all seeded PET plates were gently rinsed twice with 10 mL 37 °C pre-warmed fresh LB medium prior to being individually immersed into a new tube containing 5 mL 37 °C pre-warmed fresh LB medium at 37 °C for 1 h. After each consecutive hour of incubation one of the plates was taken out for imaging (Process (III) below), but all other remaining plates were subjected to the Process (II) again, and placed into a new fresh culture medium tube.

Process (III) – after each hour of incubation, one of the PET plates was taken out, gently washed with tris-buffered solution (TBS) thrice to remove the remains of the medium, and then incubated for 15 min in the dark with the BacLight Live/Dead viability kit (Invitrogen, kit no. L7007) to stain the cells with a fluorescent dye. Samples were then rinsed twice with TBS and immersed into a 50% glycerol-TBS solution before imaging, so that the attached cells are protected when exposed to air. The bacteria on the surfaces were visualised using Confocal Laser Scanning Microscopy (CLSM; LEICA TCS SP5). This device has an inverted optics, so that the PET plate lying flat on a clean glass slide is imaged from below (bacteria are viewed through the glass and the PET layers) with an oil-immersion objective lens at 40 × magnification, zoom 1:2.60 or 1:4.90. At least 20 fields of view were randomly chosen for analysis and the images were processed using ImageJ software (NIH, Bethesda, Maryland, http://rsbweb.nih.gov/ij/). The index j = 0, 1, …, 10 represents the number of hours bacteria have spent adhered to the surface in the refreshed culture, and n(j) describes the population of *E. coli* MG1655 on PET surfaces at each observation stage; n(0) represents the initial ‘seeded’ cells after the Process (I) completion. Each PET substrate imaged by CLSM was discarded after imaging.

Process (IV) – we also need to monitor how many bacteria have detached from the substrate at each stage of development. After each surface specimen was removed from the LB culture medium at hourly intervals specified by j, the medium incubating this PET plate was immediately diluted by phosphate-buffered solution (PBS). The bacterial concentrations (CFU/mL) in the culture media were estimated with a conventional serial dilution method with the surface spreading of the dilutions onto agar plates^28^,^29^. The agar plates were cultivated at 37 °C for 18 h; the numbers of colonies cultured from the serial dilutions were then counted and the measured counts converted to CFU/mL after multiplication with the dilution factor. The total number of cells in each 5 mL medium divided by the PET surface area (approximately 365 mm^2^ for each sample), denoted as N(j) in units of CFU/mm^2^ (with j = 1, 2, …, 10), was used to quantify the bacterial cells that detached from the surface into the external medium.

### Conventional bacterial growth curve in bulk liquid medium

For comparison between bacterial growth on surfaces and in the bulk medium, the stationary-phase synchronised *E. coli* MG1655 suspension was diluted using fresh LB medium to yield a bacterial concentration of about 1250 CFU/mL, and 100 μL of this dilution was added to 5 mL of fresh 37 °C pre-warmed LB medium. The new suspension was incubated at 37 °C, and the change in bacterial population in the medium with time (the growth curve) was measured at 1 h intervals using the conventional serial dilution method^29^, as described above.

### Furanone compound for quorum sensing inhibition

(Z-)-4-Bromo-5-(bromomethylene)-2(5 H)-furanone (Synonym: Furanone C-30, Sigma-Aldrich) was used as the quorum sensing inhibitor of *E.coli* MG1655. Furanone C-30 (FC30) was stored in ethanol at 20 mg/mL at −20 °C. In order to inhibit bacterial quorum sensing without directly killing bacteria or inhibiting their growth, MIC (minimum inhibitory concentration) was firstly determined. Briefly, bacterial suspension was diluted 1:1000 in LB as the inoculum. Twofold-dilution series of FC30 were prepared in 100 μL LB in a 96-well plate, and 100 μL of the inoculum was added. The plate was incubated for 24 h at 37 °C. MIC (minimum inhibitory concentration) is defined as the lowest concentration of FC30 at which there was no detectable growth of *E.coli* MG1655. Based on the result of MIC, twofold-dilution series of FC30 which started from half MIC were prepared in 2.5 mL LB in test tubes, and 2.5 mL of the inoculum was added. The tube for control contained 2.5 mL LB and 2.5 mL of the inoculum. These tubes were incubated for 12 h at 37 °C, and then the optical density at 600 nm (OD_600_) of these suspensions was measured. The brominated furanone dosage which was applied to incubate PET substrates would be the maximum concentration in the suspensions that showed the same OD_600_ as the control suspension. The bacterial growth on PET surfaces with the inhibition of quorum sensing was determined as illustrated in Fig. 2, and FC30 was added into the incubating and washing LB medium after 3 h.

### Statistical analysis and errors

All statistical analysis was performed using ANOVA testing within Microsoft Excel. Values were reported in the text as mean value ± standard deviation. The statistics in our observation was acquired from two sources: in each microscopic image (whether to count cells or to measure their size) the field of view contained a large number of bacteria (see Fig. 3 below for example). We have randomly chosen 20 independent fields of view for counting on each sample substrate. The whole experiment process, as illustrated in Fig. 2, has been repeated three times independently, thus making the number of samples sufficiently large and the standard deviation errors acceptable as shown in the plots below.

## Results

**Figure 1:**
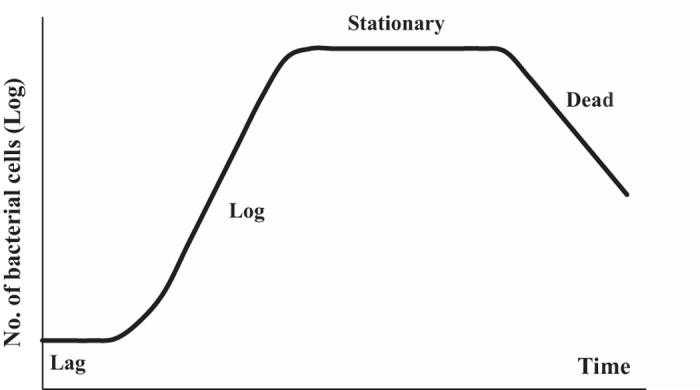
Graphic representation of typical bacterial growth curve in culture medium. When bacteria are introduced into the fresh medium in a closed system, like a test tube, the population of cells always exhibits growth dynamics as follows. Lag phase: bacteria initially adjust to the new environment, so they appear not able to replicate but the cells might grow in volume; Log (exponential) phase: cells start dividing regularly by the process of binary fission. The culture reaches the maximum growth rate, which could be estimated by generation time or doubling time, i.e., the time per generation; Stationary phase: the number of cells undergoing division seems to be equal to that being dead due to the exhaustion of nutrients. Dead phase: bacteria lose the ability to divide and the number of dead cells exceeds that of live cells.

**Figure 2:**
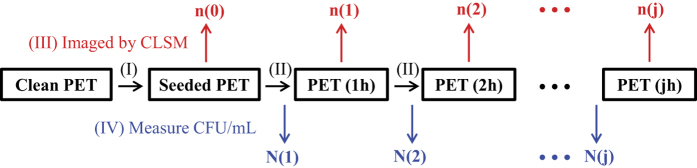
Schematic diagram illustrating the procedure to measure the amount of bacterial cells on PET surfaces per mm^2^ (i.e. n(j), j = 0, 1, 2, …) and the number of cells that detach from surfaces into culture medium (i.e. N(j), j = 1, 2, …) at each hour interval. The labeled Process (I, II, III or IV) is described in details in Methods.

**Figure 3:**
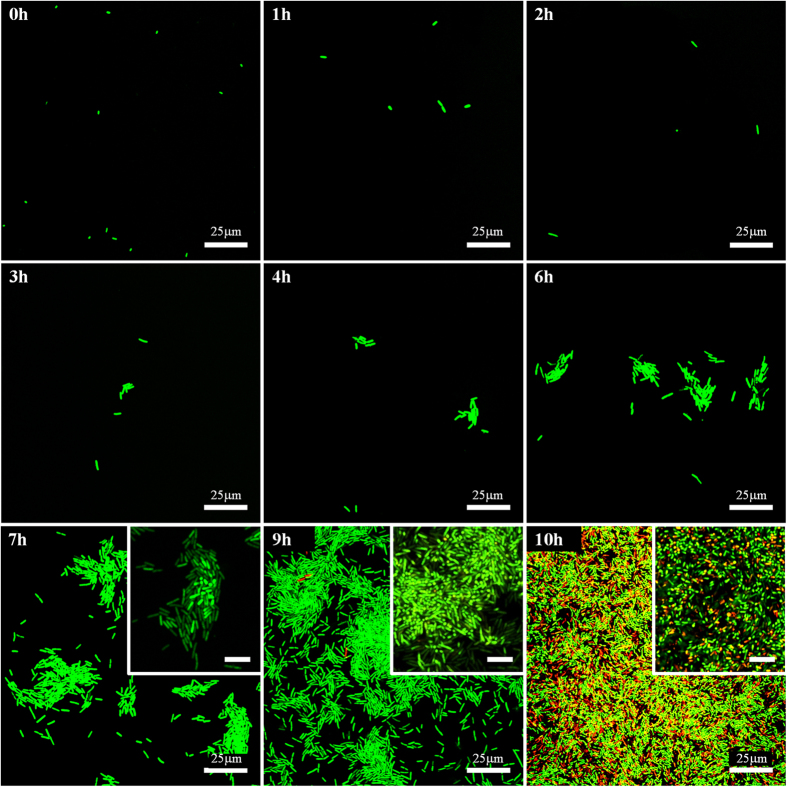
CLSM fluorescence images of *E. coli* MG1655 cells on PET surfaces at each hour interval. The insets of 7–10 h show the upper layer of cells in the multilayer colony, when they are put in focus (CLSM was conducted with 3D-scanning-mode), inset scale bar: 10 μm. Live and dead cells are stained with green and red, respectively.

**Figure 4:**
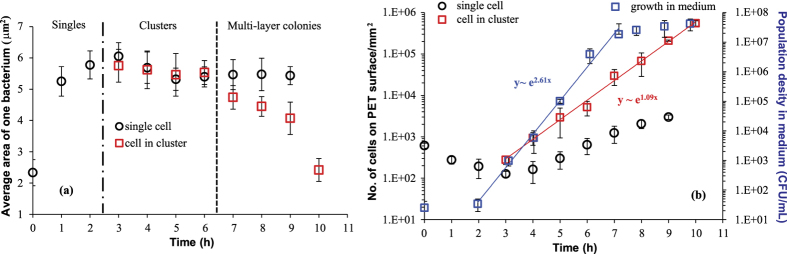
(**a**) The cell size of bacteria on PET surfaces at each hour interval. (**b**) The curves of bacterial growth on PET surfaces and in a separate test in LB bulk culture medium (for comparison). The exponential regression fits in the log phase are shown for the bulk and substrate populations. Singles are no longer measured at 10 h since it is difficult to distinguish the single cells from the cells in clusters then.

The CLSM images of PET substrates removed from the external culture medium at each hour interval (Fig. 3), and the change of the average individual cell size with time, obtained from these fluorescent images, (Fig. 4a) outline the three stages of bacterial evolution on PET surfaces after the initial adhesion:

- Over the first 2 hours, the adhered bacteria do not replicate, as only single cells are seen on surfaces. In the meantime, we could tell that cells remaining on the surface dramatically increased in size, especially during the first hour. Hence, this stage could be designated as the lag phase of bacterial growth on PET surfaces, which differs slightly from the usual ‘flat’ lag phase in the bulk medium, described in Fig. 1: in the first few hours the amount of adhering cells decreased with incubation time since some of the initially attached cells have left the surface into the fresh medium. This is because the initial bacterial adhesion is reversible with a low barrier for desorption^6^.
- After the substrates were cultivated in LB medium for about 3 hours, the bacteria on the surface began to divide with the daughter cells spreading outward to form small one-layer thick cell clusters. This represents the beginning of bacterial colonisation on the substrate. From 3 to 6 h, more one-layer clusters of increasing size were observed but the sizes of individual cells did not show any significant change for either single or clustered bacteria.
- By 7 to 10 h, in parallel with the continuing development of bacterial clusters, multiple layers of cells in biggest clusters became detectable within the micro-colonies. At around 10 h the entire surface area was occupied by these multi-layered colonies and a host of dead cells could also be seen. It is noteworthy that in this phase the average size of each cell in the multi-layer colonies underwent a clear decrease as colonisation progressed, whilst the single cells (when they could be observed) maintained the same size as in the stage (ii). We return to this issue in detail in the Discussion.

The average cell density (n(j), for j = 0, 1, 2, …) obtained from the fluorescent images is presented in Fig. 4b for the quantitative analysis of the bacterial population on PET surfaces during incubation (the control experiment with the growth curve of *E. coli* MG1655 in the bulk LB medium is also shown in this plot for comparison). The log-linear plot emphasizes the exponential growth of bacteria in clusters that appeared after the lag phase (the error bars here, and in other plots, are the standard deviation calculated from the large number of independent measurements at each data point). Therefore, the stage of (ii)–(iii) could be regarded as the log phase of bacterial growth on PET surfaces. External nutrients were plentiful in our study (the medium refreshed every hour), and the exponential proliferation in clusters on surfaces is qualitatively the same as that of the log phase in a bulk medium.

Exponential regression analysis showed that the specific growth rates (α) for bacterial growth on PET surfaces and in the bulk liquid culture were 1.09 and 2.61 h^−1^, respectively. Thus the corresponding generation (doubling) times τ would be approximately 38 min and 16 min, respectively. The bulk value of doubling time corresponds well to the literature data^30^. Why was the doubling time of the sessile bacteria on substrates more than double than that of planktonic cells (a term referring to free-swimming bacteria) in the same culture liquid? One possibility is that the bacteria needed more time to replicate at the solid-liquid interface, because only about half of their surface area could absorb the surrounding nutrition; at the same time, they needed to work harder to use their appendages to remain at the surface. Another reason might be the detachment of the daughter cells back into the bulk medium, which reduces the apparent count n(j). Hence, the detachment of cells from surfaces over all three stages was investigated.

**Figure 5:**
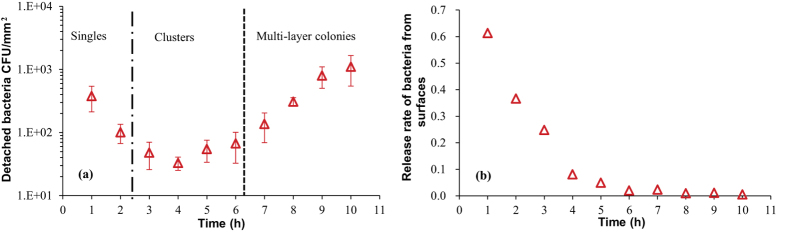
The dynamics of bacterial detachment from PET surfaces during the whole growth. (**a**) The absolute number of detached cells, N(j). (**b**) The rate of detachment β(j), plotted against time (at each hour interval).

The density N(j) (j = 1, 2, …, 10) represents the amount of bacteria in the culture medium at the end of each one-hour interval of incubation in the fresh medium. Since the generation time in the bulk is only 16 min, some of the detached bacteria could have replicated several times in the one hour of incubation, so we first determined whether the detached cells divided during the interval. In the phase (i), that is for j = 1, 2, no bacterial replication was observed on PET surfaces, thus Δn(1) = n(0) – n(1) = 339 cells/mm^2^ and Δn(2) = n(1) – n(2) = 83 cells/mm^2^ would be the exact number of detached cells during the first and second intervals. As seen in Fig. 5a, the values of N(1) and N(2) were 377 and 101 cells/mm^2^ respectively, indicating that almost none of the detached cells divided in the culture medium during each interval. In fact, this is consistent with the necessary lag phase after a new cell detachment. In the exponential growth stage on the substrate, even with a slight overestimation of the number of these detached cells respectively added to n(j) (j = 3, 4, …, 10) during each hour, the refreshed specific growth rate on the surface (α ≈ 1.05 h^−1^) did not significantly change. We may thus conclude that the detachment of the daughter cells was not the reason for the slower bacterial growth on PET surfaces.

As demonstrated in the plots of N(j) and n(j) with increasing incubation time j, the dynamics of detachment appears to depend on the bacterial density on the surface. Thus, we introduce a parameter β(j), again a function of time measured by j = 1, 2, …, 10, to roughly describe the proportion of cells that detached from the surface at each interval (one may call it the ‘release rate’):


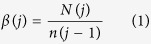


It could be seen in Fig. 5b that the release rate is not constant; β(j) sharply decreased with time when only single cells on surfaces were observed, in stage (i). We interpret this as the result of gradually increasing the attachment strength of individual cells as they settle on the surface (we discuss this in greater detail later in the text). Once the cell clusters grew into larger micro-colonies, the rate of detachment β(j) varied much less (although continued to decline slowly). We interpret this as increasingly strong adhesion of bacteria to their neighbours as well as to the surface. Although the gross amount of detached cells increased with the development of micro-colonies, after 4 h, the fraction of sessile cells released into the medium slowly decreased. For incubation longer than 4 h, the overly large n(j) caused β(j) to converge towards zero. This finding seems to demonstrate that the colonised bacteria were more likely to remain on the surface with the progressing colonisation.

## Discussion

The results clarify the growth curve pattern of *E. coli* on PET surfaces and reveal some details of bacterial life after their adhesion onto surfaces in the presence of abundant externally supplied nutrients. This growth curve appears to exhibit distinct phases: the lag and the exponential (log) phases, as with the conventional growth curve for bacteria in a bulk liquid medium.

At time point zero, the ‘seeded’ cells on the surface were the result of bacterial adhesion when the clean surface was immersed in the stationary-phase synchronised bacterial cells suspension. Usually this adhesion is described in two separate stages: ‘reversible’ and ‘irreversible’ adhesion^6^,^10^. Over the first 2 h, in stage (i), the reversibly adherent bacteria tended to detach from the surface because the activation energy to desorb was low and the cells might have preferred a free-swimming or planktonic lifestyle in the fresh culture medium, resulting in a decrease in the number of sessile cells. It is unclear whether the rest of cells on the surface after 2 h became irreversibly attached, but they must have had a stronger bacteria-surface bond with surfaces to remain immobilised, possibly because the appendages of bacteria (fimbriae or pili) overcome the physical repulsive forces of the electrical double layer^31^, and stimulate the contact between the cells and the surfaces. In addition to the adjustment of the number of bacteria and the bond strength with surfaces, the dimensions of the individual cells on PET surfaces also increased significantly after cultivation in a new environment, similar to the bacterial behaviour during the lag phase in a bulk medium. In this stage, the cells that remain on the surface are metabolically active and synthesise the enzymes and factors needed to enter the next stage of cell division^32^. Note that the duration of the lag phase turns out to be the same for bacteria on the surface and the planktonic ones in the bulk of the same medium, as is clear from Fig. 4b.

After the lag phase, an exponential growth of forming cell clusters was observed, stages (ii)–(iii). As the cells divided, the daughter cells spread outward and upward into clusters rather than occupying the entire surface area in a monolayer, which is consistent with the literature observations^13^. At the same time, we noticed that the amount of single cells also increased over time (j ∈ [3, 9]; it was difficult to distinguish the single cells from the cells in clusters at 10 h). So what is the source of the greater numbers of single bacteria during the later stages of many dense micro-colonies? One possibility might be the recruitment of planktonic cells from culture medium^33^. However, in this study we refreshed the medium at each hour, so the density of detached cells during the interval only reached ~10^3^–10^4^ CFU/mL at maximum, which is very much lower than the bacterial concentration in the stationary-phase synchronised suspension (~10^9^ CFU/mL) during our original surface deposition. In that process of initial ‘seeding’, only around 600 cells/mm^2^ could be detected on the surface; therefore, the recruitment from a much more diluted medium seems unlikely to have contributed a noticeable amount of single cells on the surfaces. Another possibility is the migration or redistribution of the replicating cells on the substrate, which might be assisted by the motility of *E. coli* mediated by flagella and type 1 pili^34^.

During the exponential growth phase, the bacterial generation time on PET surfaces was found to be much longer than that of free growing bacteria in the same culture medium. The results for the detachment over this period proved that this longer doubling time was not due to a loss of daughter cells from the surfaces into the medium, but instead to the less efficient cell division of sessile bacteria compared with that of planktonic cells in culture medium. This is not difficult to understand, since in addition to the process of cell replication, some intracellular adenosine triphosphate (ATP) must be spent on bacterial anchoring at the interface; for instance, the assembly of fimbriae that aid in bacterial adhesion is at the expense of an energy cost^35^. At 10 h and later, a large amount of dead cells were observed on the surface. In the traditional analysis of bacterial growth in bulk medium, dead cells are thought to appear because of the exhausted nutrients and the accumulation of waste materials, toxic metabolites and inhibitory compounds. In our study this was different: the incubation medium was refreshed every hour, so that sufficient quantities of nutrients were always available during growth, and toxin accumulation avoided. In spite of that, we still saw the dead cells appearing prominently after 10 h of incubation. This suggests that the high density of cells alone might trigger the cell death, irrespective of the presence of toxins or starvation, and may be the start of the stationary phase of the growth curve.

The dynamics of detachment was expected to just be related to the number of cells on surfaces. However, the number of detached cells at 8 h was nearly same as that at 1 h, but there were almost 50 times as many cells on PET surfaces at 7 h than initially adhered ones. It indicates that the detachment or release of bacteria from the surface depends also on the living status (single/clustered) of the adherent cells. The low barrier of desorption in the lag phase makes reversibly adherent single cells easy to detach. After bacteria adjust themselves to proliferation on surfaces, their adhesive strength enhances and they tend to remain on the surface with the somewhat irreversible adhesion. It could be demonstrated that single cells in the lag phase have greater potential for detachment than colonised cells. With the development of micro-colonies, the bacteria-surface bond strengthens and the activation energy to desorb from surfaces thus seems to increase, therefore fewer fractions of sessile cells would detach.

The average intracellular concentration of FtsZ for *E. coli*, was found to remain constant over the course of the cell cycle^36^,^37^. The total amount of FtsZ in individual *E. coli* cells would therefore increase with the cell size. As discussed in the Introduction, the accumulation of FtsZ has to reach a sufficient level to initiate cell division, which explains why the *E. coli* cells in the lag phase delay division until they have achieved a size with sufficient levels of FtsZ to replicate on surfaces.

It is worth noting that the LB broth used to cultivate bacteria in our study is a carbon-rich complex medium. Based on the nutrient-dependent mechanism also mentioned in the Introduction, the size of daughter cell in this study is supposed to be larger during the log phase (around 5.5 μm^2^ for each cell area in the beginning of the log phase claimed in Fig. 4a) than usual *E. coli* size (0.5 × 3 μm): the nutrient-rich LB medium leads to a high activity of the FtsZ inhibitor^18^,^22^,^37^. The inhibition would reduce the amount of assembly-competent FtsZ used to form the Z ring at the end of the cell cycle; thus, the bacterial cell has to increase its size to reach a sufficient level of assembly-competent FtsZ, which enlarges the daughter cell size.

The size of the remaining single bacteria on PET surfaces was maintained at this high level over the entire log phase and did not show any obvious change. In the initial log phase (j ∈ [3, 6]), no difference were apparent between the sizes of the individual cells in clusters and those in single form. However, after the formation of multiple layers of cells, the average area of one cell in the micro-colonies decreased rapidly over time (j ∈ [7, 10]). Over this period, the differences between the sizes of individual cells in multi-layer micro-colonies and those in singles were significant. Specifically, for our 25 independent measurements, setting α = 0.05 in ANOVA, we obtain P = 0.037, 0.001, 0.001, and 1.1E-09 at 7 h, 8 h, 9 h and 10 h, respectively. I.e. in all cases P < 0.05, which implies that the size control of the individual cells involved in bacterial exponential growth on PET surfaces is not modulated by time but by the density of bacterial cells in clusters or micro-colonies. Athale *et al.* reported that the percentage of long cells decreased as the cell density increased when bacteria were grown in bulk medium; they considered this phenomenon to be due to a result of lower nutrient availability^38^. However, in our study, the cells on the surfaces were supplied with abundant nutrition, and the dynamics of growth on PET surfaces also showed a steady growth rate during 3–10 h. Therefore, the limitation due to nutrient availability does not seem applicable in our study. What, then, leads to the decrease in the size of clustered cells after they are in dense density? The cell size of *E. coli* MG1655 growing on PET surfaces is therefore thought to be controlled by a density-dependent metabolic pathway, which is often referred to as ‘quorum sensing’.

**Figure 6:**
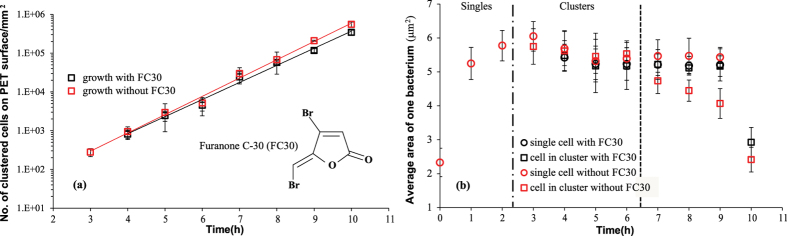
Influence of quorum sensing inhibitor Furanone C-30 (FC30) on (**a**) bacterial growth and (**b**) bacterial cell size on PET surfaces. The chemical structure of Furanone C-30 is shown in the inset of (**a**). The red data in (**a**,**b**) comes from Fig. 4a,b, respectively (i.e. reference without quorum sensing inhibition).

In order to investigate the mechanism of bacterial size control and the emergence of the 3D (multilayer) structure, we studied the effect of added quorum sensing inhibitor (the signal antagonist) brominated furanone (FC30). There are many reports of natural brominated furanones and derivatives inhibiting the quorum sensing^39^,^40^,^41^. In gram-negative bacteria, the brominated furanones compete with the quorum sensing signal molecules for a common binding site on LuxR-type transcriptional activators, which are responsible to regulate expression of target genes for the subsequent quorum sensing circuit^41^,^42^,^43^,^44^. However, they also inhibit the cell growth at higher concentration. In fact, we found that the growth was completely stopped at the MIC of 50 mg/L. Hence we first had to determine FC30 concentration in the medium at which the growth rate was un-inhibited, which was found in our case to be below 1.56 mg/L. Figure 6(a) confirms that the overall rate of growth on substrate was not altered by the addition of FC30 at this low concentration. Comparing the cell size in the samples with the inhibitor FC30 and without it (as in Fig. 4a before), we find in Fig. 6b that the marked decrease in size we saw earlier does not happen with quorum sensing inhibition. Instead, the bacteria in clusters retain their large size as they had as singles up to 9 h of growth on the surface, while with no inhibition – the size decrease started between 6 and 7 h.

**Figure 7:**
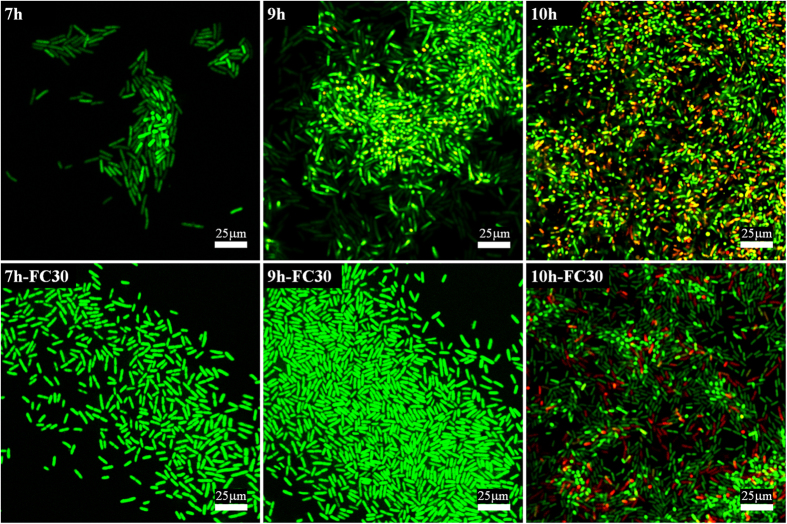
The effect of quorum sensing inhibitor Furanone C-30 (FC30) on the micro-colonies morphology of *E. coli* cells on PET surfaces after 7–10 h incubation. The first row comes from the inset images from Fig. 3 (i.e. reference without quorum sensing inhibition), while the second row shows cell clusters growing with Furanone C-30. Live and dead cells are stained with green and red, respectively.

Figure 7 illustrates the difference in colony morphology with the quorum sensing suppressed. The top row shows the inset images from the earlier Fig. 3. We see that the 3D structure appears prominent already at 7 hours of incubation in reference samples, as shown by confocal plane focusing on the higher layer in cell clusters above the surface. In contrast, with FC30 added to the culture medium, the colonies remain flat on the surface. These results indicate that the quorum sensing system might regulate the cell size control and the emergence of 3D structure of biofilm. Only at 10 h of incubation do we start seeing a few bacteria emerging in the second layer. Note that at 10 h the size of bacteria in Fig. 6b was also seen to rapidly decrease. Clearly at this concentration of cells on the surface the quorum sensing signal molecules finally overwhelm the FC30 quorum sensing inhibition and the multi-layer colony growth recovers.

**Figure 8:**
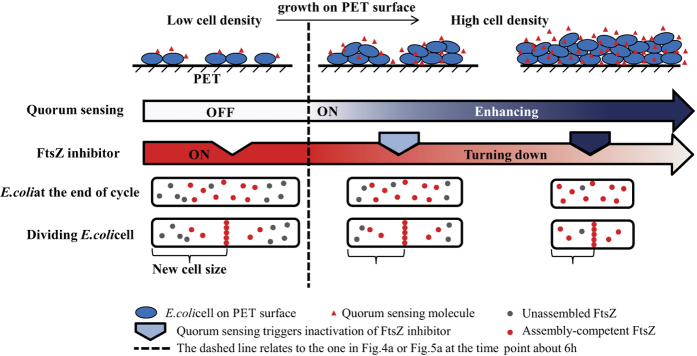
The *E. coli* cell size control model when growing on PET surfaces.

The quorum sensing-regulated cell size on PET surfaces could be therefore illustrated as the right part of Fig. 8: First, bacteria produce and release signal molecules that increase in concentration as a function of cell density. After the concentration of these signals within the bacterial micro-colonies reaches a threshold, the cells are able to recognise their high population^45^,^46^. The proportional quorum sensing then seems to stimulate proportional inactivity of the FtsZ inhibitor, thus a greater fraction of FtsZ in the cell contributes to the FtsZ ring with the progressing colonisation, which means that the bacteria on the surface can divide with sufficient assembly-competent FtsZ proteins at a smaller size. Subsequently, the size of the daughter cells would decrease with the increase of bacterial density on PET surfaces. The size of the individual cells in single form remained unaltered during the entire growing process in this study, because the quorum sensing did not take place amongst the single cells.

## Conclusions

This study reveals that in the presence of sufficient rich nutrients, the microbial-contaminated surfaces of medical devices or food containers would be infected by heavy micro-colonies with an exponential growth on the surfaces. The cells in the micro-colonies have stronger adhesive bonds with the substrates as bacterial clusters develop, which means that the clustered bacteria would be hard to remove from surfaces with the progressing colonisation. Over the bacterial growth on PET, quorum sensing is found to play an important role in controlling the cell size.

## Additional Information

**How to cite this article**: Wang, L. *et al.* Bacterial growth, detachment and cell size control on polyethylene terephthalate surfaces. *Sci. Rep.* **5**, 15159; doi: 10.1038/srep15159 (2015).
